# Occurrence and Characterization of the Pathogenic Potential of *Bacillus cereus* in Vending Machines from Southern Italy

**DOI:** 10.3390/foods15183296

**Published:** 2026-09-17

**Authors:** Mario Astuni, Marina Ceruso, Lucia Anella Ammaturo, Giovanni Battista Varcasia, Iolanda Venuti, Marta Castrica, Tiziana Pepe

**Affiliations:** 1 Department of Veterinary Medicine and Animal Production, University “Federico II”, 80137 Naples, Italy; mario.astuni@unina.it (M.A.); luciaanella.ammaturo@unina.it (L.A.A.); tiziana.pepe@unina.it (T.P.); 2 A.S.L. Napoli 1 Centro (Azienda Sanitaria Locale), 80145 Naples, Italy; giovannibattista.varcasia@aslnapoli1centro.it (G.B.V.); iolandavenuti@gmail.com (I.V.); 3 Department of Life Sciences, Health and Health Professions, Link Campus University, 00165 Rome, Italy; m.castrica@unilink.it

**Keywords:** foodborne pathogens, virulence genes, food safety, food hygiene

## Abstract

*Bacillus cereus* is a ubiquitous spore-forming bacterium associated with foodborne illness. Although frequently detected as a contaminant in food-processing environments, the presence of specific virulence-associated genes may be relevant to food safety. Pathogenicity is mainly associated with production of emetic and diarrheal toxins. In a previous survey assessing the hygienic and sanitary conditions of vending machines in the Campania Region (Italy), *B. cereus* was the predominant pathogenic microorganism recovered. The present study aimed to characterize the virulence-associated gene profiles of DNA extracts from these *B. cereus*-positive samples to assess their potential pathogenicity-related and food-safety relevance. A total of 221 samples, including finished products (cappuccino), powdered ingredients (e.g., milk and coffee), and surface swabs, were collected. *B. cereus* was detected in 51.6% (114/221) samples using the *nhe* gene for species identification. The virulence-associated genes *cwpFM*, *cytK*, *hblD*, and *ces*, among the most frequently associated with foodborne illness, were investigated by end-point Polymerase Chain Reaction (PCR). The prevalence was 67% for *cwpFM*, 54% for *cytK*, 53% for *hblD*, and 11% for *ces*. These findings reveal virulence- and persistence-associated genetic markers in *B. cereus*-positive vending machine samples, highlighting the need for effective hygiene measures, improved sanitation, and enhanced monitoring.

## 1. Introduction

*Bacillus cereus* (*B. cereus*) is a ubiquitous, Gram-positive, spore-forming bacterium widely distributed in natural and food-processing environments. The capacity of *B. cereus* to produce highly resistant endospores and form robust biofilms contributes significantly to its persistence under adverse environmental conditions, promoting recurrent contamination throughout the food production chain, including raw materials, processing equipment, and ready-to-eat foods [1,2]. Owing to its remarkable ability to persist throughout the food chain, *B. cereus* is one of the most prevalent microbial contaminants of food products and food-contact surfaces.

The presence of *B. cereus* in food represents not only a hygienic quality concern but also a significant food safety hazard. It is estimated to be responsible for 1.4% to 12% of foodborne outbreaks worldwide and consistently ranks among the leading bacterial agents of foodborne illness in the European Union [3,4]. Although historically associated with mild, self-limiting gastroenteritis, *B. cereus* is now recognized as an opportunistic pathogen capable of causing severe and occasionally fatal infections [4].

The pathogenicity of *B. cereus* is closely associated with the presence of virulence genes encoding enterotoxins and other pathogenicity-related factors. Numerous studies have demonstrated considerable heterogeneity in the distribution of these genes, indicating that not all strains pose the same risk to consumers [5]. Consequently, molecular characterization of virulence-associated genes has become essential for assessing the pathogenic potential of *B. cereus* strains circulating in the food chain.

Gastrointestinal disease caused by *B. cereus* is classically divided into two syndromes. The emetic form results from ingestion of the preformed, heat-stable toxin cereulide, produced in food and encoded by the plasmid-borne *ces* gene cluster. The diarrheal form is a toxico-infection caused by enterotoxins produced in the small intestine following ingestion of viable cells or germinating spores. These toxins include the non-hemolytic enterotoxin (nhe), hemolysin BL (hbl), and cytotoxin K (cytK) [4].

Biofilm formation is a key ecological strategy that contributes to the environmental persistence of *B. cereus* [6]. Mature biofilms, particularly those formed on stainless steel surfaces, may contain up to 90% differentiated endospores [7]. The extracellular biofilm matrix protects these endospores from environmental and chemical stresses, enhancing their long-term survival and making biofilms persistent reservoirs of contamination [6,7]. Furthermore, the capacity to form biofilms varies considerably among strains within the *B. cereus* group, with notable differences observed between pathogenic and non-pathogenic isolates [8]. Among the factors involved in biofilm development, the cell wall-associated peptidase cwpFM (formerly entFM) plays a central role. This multifunctional protein contributes to cell wall remodeling, adhesion, motility, and biofilm formation [9]. Furthermore, recent studies aimed at improving the discrimination between pathogenic and non-pathogenic *B. cereus* strains have identified intrinsically disordered regions within cwpFM, particularly the IDL4 segment. This region was found to be associated with pathogenic strains and absent in non-pathogenic isolates, suggesting that cwpFM may contribute not only to environmental persistence but also to bacterial virulence [5].

Automated food-dispensing systems, such as hot-drink vending machines (VMs), represent favorable ecological niches for *B. cereus*. Internal components, including water pipes, mixing chambers, and dispensing nozzles, provide abiotic surfaces that facilitate bacterial adhesion and biofilm formation [10,11]. Their structural complexity, combined with intermittent sanitation procedures, further promotes persistent contamination [11,12]. In addition, powdered ingredients commonly used in VMs, such as milk powder and coffee, are characterized by low water activity, which inhibits vegetative growth while allowing long-term survival of bacterial endospores. During beverage preparation, changes in temperature and water activity may trigger spore germination, allowing the outgrowth of vegetative cells and subsequent toxin production under favorable conditions.

In a previous survey evaluating the hygienic and sanitary conditions of VMs in the Campania Region (Italy) [12], *B. cereus* was the pathogenic microorganism consistently detected in all VMs examined. These findings highlight the need for further investigations into the food safety relevance of products dispensed from VMs systems. In particular, the combined evaluation of virulence- and persistence-associated gene profiles in *B. cereus*-positive samples from VM environments remains poorly investigated despite the widespread use of these systems and their potential relevance for consumer exposure [13].

Accordingly, the present study aimed to characterize virulence- and persistence-associated gene profiles in *B. cereus*-positive samples collected from VM products and components by investigating genes associated with diarrheal toxins (*cytK* and *hblD*), the emetic toxin (*ces*), and biofilm formation (*cwpFM*), in order to evaluate their potential pathogenicity-related and food-safety relevance.

## 2. Materials and Methods

### 2.1. DNA Samples

The present study focused on the molecular characterization of DNA extracts obtained from *B. cereus*-positive samples previously identified during a survey assessing the hygienic conditions and microbiological safety of VMs in the Campania Region, Italy [12]. The original investigation was conducted between October 2021 and February 2023 and involved 30 hot-drink VMs located in different settings, including universities, schools, hospitals, and workplaces. Notably, *B. cereus* was detected in at least one sample from 25 of the 30 VM examined.

**Figure 1:**
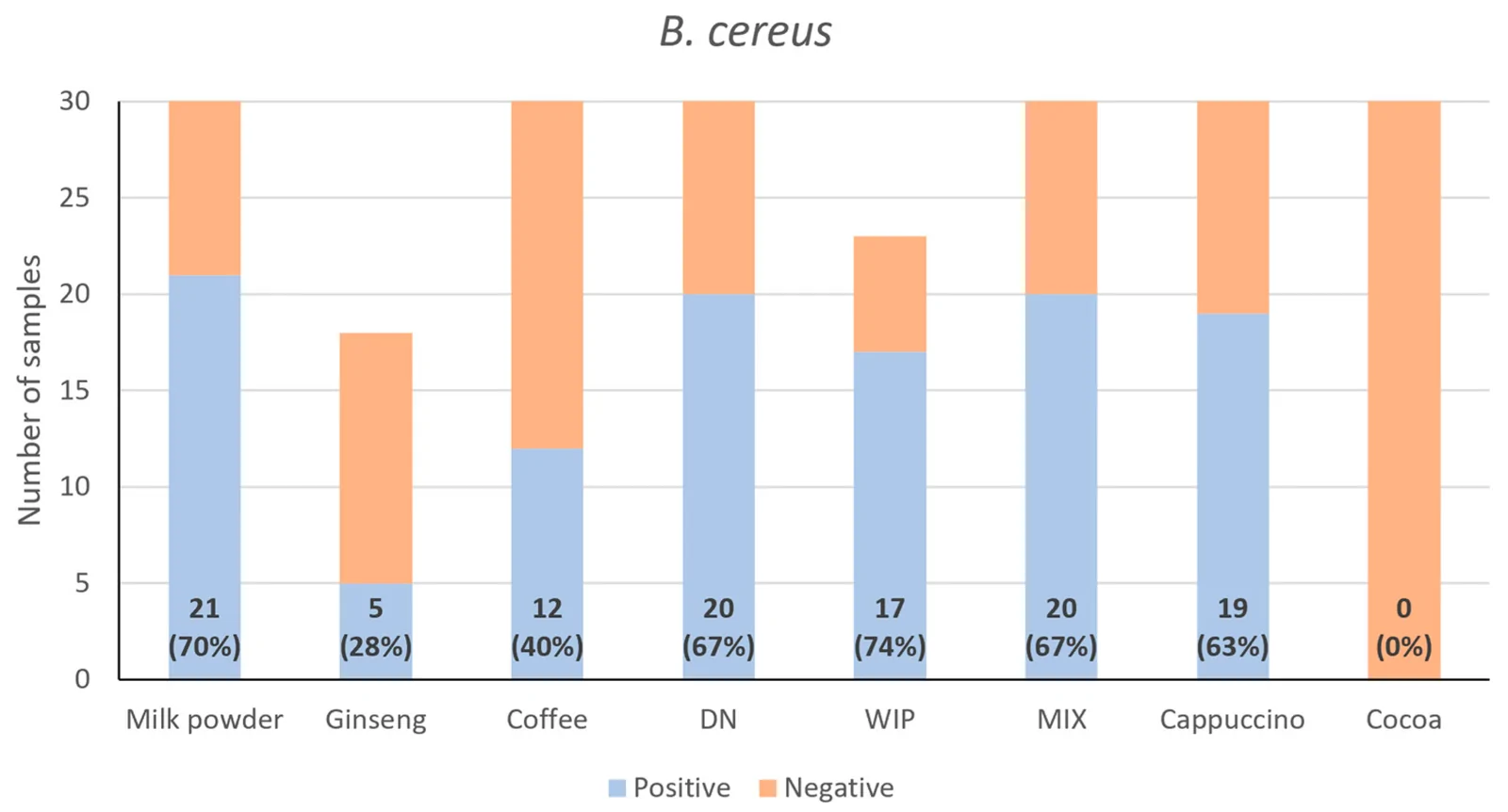
Distribution of *B. cereus*-positive samples by product and sample category. Bars represent the number and percentage of positive (blue) and negative (orange) samples for the following matrix: Milk powder, Ginseng, Coffee, Dispensing Nozzle (DN), Water Intake Pipes (WIP), Mixer bowls (MIX), Cappuccino, Cocoa.

A total of 221 samples were collected, comprising swabs from internal VM components (water intake pipes, mixing chambers, and dispensing nozzles), powdered ingredients (milk powder, coffee, cocoa, and ginseng), and dispensed hot beverages (cappuccino). Overall, 114 samples (51.6%) tested positive for *B. cereus* (Figure 1). DNA extracts obtained from these positive samples were preserved and subsequently used for the molecular analyses performed in the present study. No additional sampling, bacterial isolation, or DNA extraction procedures were carried out.

Supplementary Table S1 provides detailed information on the sampling scheme, including the specific Vending Machine ID, the number of samples collected, the number of positive results subjected to PCR, and the relative virulence gene profiles.

### 2.2. DNA Extraction and Quality Assessment

Genomic DNA had been previously extracted from enriched VM samples using the DNeasy Blood and Tissue Kit (Qiagen, Hilden, Germany), following the manufacturer’s protocol for Gram-positive and Gram-negative bacteria. DNA was eluted in the final volume of 50 µL of sterile distilled water. DNA quality and purity were evaluated using the MaestroNano Pro device (MaestroGen Inc., Taiwan, China), and only samples with A260/A280 ratios between 1.8 and 2.0 were included in analyses. DNA extracts were stored at −80 °C until molecular characterization.

### 2.3. B. cereus-Positive Samples Distribution

Only DNA samples previously confirmed as *B. cereus*-positive by qualitative SYBR Green real-time polymerase chain reaction (PCR) were included in this study. These DNA extracts constituted the analytical dataset for virulence and persistence gene profiling. Positive samples were identified in powdered ingredients (milk and ginseng), internal surfaces (water intake pipes (WIP), mixer bowls (MIX), dispensing nozzles (DN), and finished beverages (cappuccino), suggesting multiple contamination routes within the vending system. The distribution of *B. cereus*-positive samples across different sample categories is shown in Figure 1.

### 2.4. PCR Endpoint and Primers Specificity

PCR amplifications were performed in a final reaction volume of 25 µL, using 12.5 µL MyTaq mix (Bioline, London, UK), 1 µL of each forward and reverse primer, 1 µL of DNA template, and molecular biology water (BioFroxx, Guangzhou, China). Reactions were performed using a 2720 Thermal Cycler (Applied Biosystems, Thermo Fisher Scientific, Waltham, MA, USA) under the following protocol: initial denaturation at 95 °C for 5 min, followed by 30 amplification cycles of 15 s at 95 °C (denaturation), 10 s at 58 °C (annealing), 45 s at 72 °C (extension), and a final extension step for 5 min at 72 °C. Positive and negative controls were included in each PCR run to ensure amplification specificity and to exclude contamination. The reference strain *B. cereus* ATCC 14579 was used as a positive control for the *cytK*, *hblD*, and *cwpFM* genes, whereas a field isolate previously confirmed by our laboratory to carry the *ces* gene served as the positive control for the emetic toxin determinant.

Before DNA extraction, the positive control strains were cultured on Trypticase Soy Agar (TSA, Oxoid, Wesel, Germany) for 24 h at 32 °C. Subsequently, a single colony from each strain was inoculated into 5 mL of Brain Heart Infusion broth (BHI, Oxoid, Basingstoke, UK) and incubated at 30 °C for 48 h; Nuclease-free water was used as a negative control.

The virulence- and persistence-associated gene profiles of the *B. cereus*-positive samples were assessed by endpoint PCR targeting genes encoding major virulence and persistence determinants, including the diarrheal toxin gene *cytK*, the hemolysin BL component gene *hblD*, the emetic toxin synthetase gene *ces*, and the biofilm-associated cell wall peptidase gene *cwpFM*. Primer sequences and amplification conditions are reported in Table 1.

#### Agarose Gel Electrophoresis and Visualization

PCR amplification products were analyzed by electrophoresis on 2% agarose gels stained with ethidium bromide and visualized via ultraviolet transillumination with Universal Hood II Gel Doc System (Bio-Rad, Hercules, CA, USA). The 100 bp DNA ladder (Solis BioDyne, Tartu, Estonia) was used as a molecular size marker.

### 2.5. DNA Sequencing of PCR Amplicons

To further verify the identity and specificity of the PCR products, representative amplicons obtained from the analyzed samples for the target genes investigated by endpoint PCR were subjected to DNA sequencing. A total of 20 amplicons were sequenced, 5 for each target gene (Table S1).

The PCR products were sent to Biofab Research (Rome, Italy) for Sanger sequencing, and the obtained sequences were compared with reference nucleotide sequences available in the NCBI database using the BLASTn algorithm (BLAST+ 2.17.0). For all analyzed amplicons, the obtained sequences showed 98–100% nucleotide identity and 98–100% query coverage with the corresponding *B. cereus* reference sequences, confirming the identity of the PCR amplicons and providing independent support for the specificity of the PCR amplification.

### 2.6. Statistical Analysis

Statistical analyses were carried out using IBM SPSS Statistics software (V.30.0); Cochran’s Q-test was applied to identify significant variations in the detection frequencies of the investigated virulence and persistence genes, while gene co-occurrence was evaluated using Fisher’s exact test. To further explore the relationship between the virulence-associated gene profile of the analyzed samples and their matrix of origin, each sample received a cumulative ‘virulence-associated gene score’ (ranging from 0 to 4, corresponding to the number of detected genes); the *nhe* gene was excluded from this score calculation, as its baseline detection was the preliminary inclusion criterion for the 114 samples analyzed in this study. The non-parametric Kruskal–Wallis H test was subsequently used to compare these virulence scores across the seven sampled matrices. A *p*-value < 0.05 was established as the threshold for statistical significance.

## 3. Results

### 3.1. Distribution of Virulence Genes

**Figure 2:**
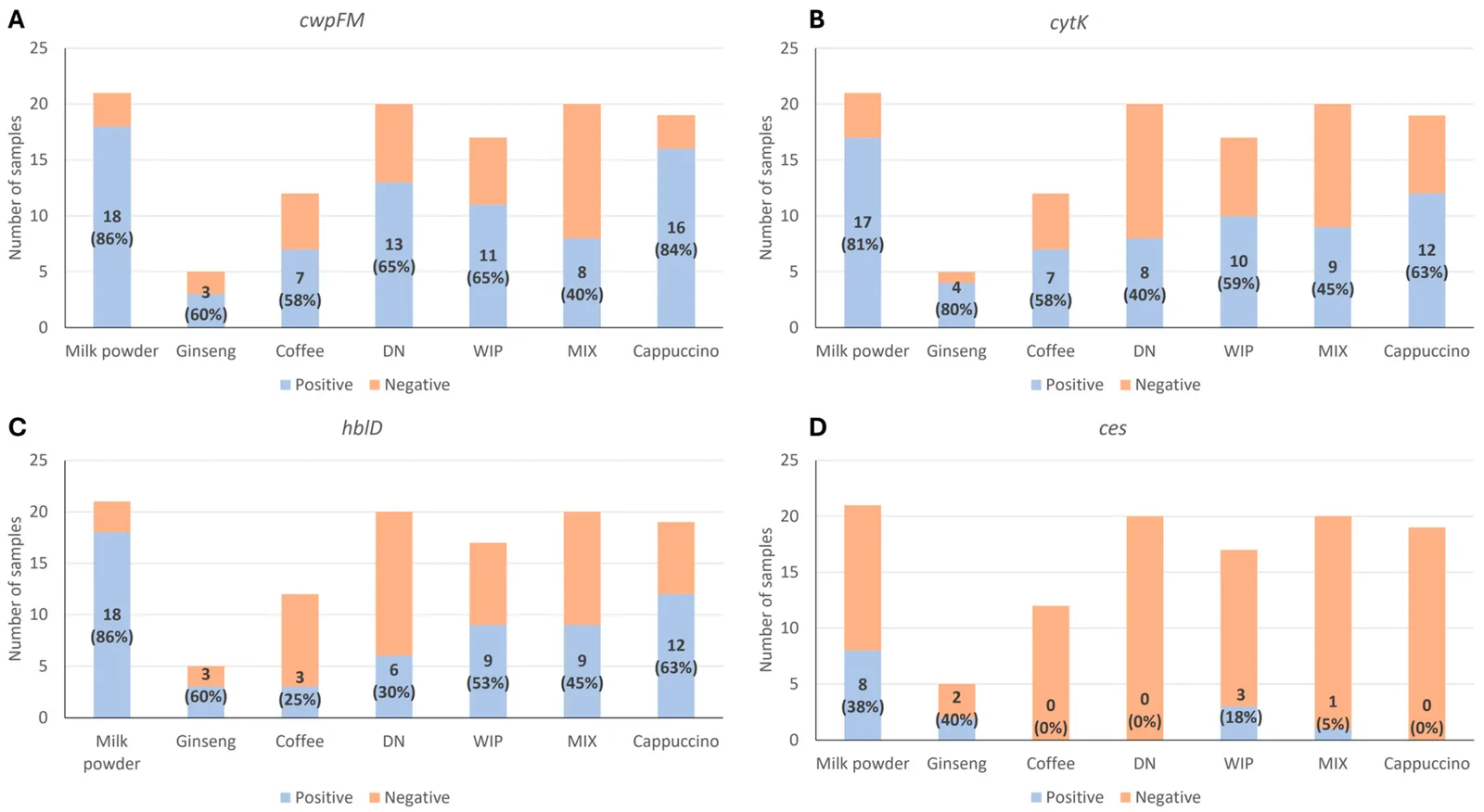
Distribution of virulence- and persistence-associated genes detected in *B. cereus*-positive samples from different VMs product categories. Panels represent the detection of: (**A**) *cwpFM*, (**B**) *cytK*, (**C**) *hblD*, and (**D**) *ces*. Bars represent the number and percentage of positive (blue) and negative (orange) samples for the following matrix: Milk powder, Ginseng, Coffee, Dispensing Nozzle (DN), Water Intake Pipes (WIP), Mixer bowls (MIX), Cappuccino.

The frequencies of virulence- and persistence-associated genes varied significantly among the 114 *B. cereus*-positive samples (Cochran’s Q test, *p* < 0.001); the most prevalent determinant was the biofilm-associated gene *cwpFM,* with a presence in 76/114 samples. It was followed by enterotoxin genes *cytK* with 61/114 positive samples and *hblD* with 60/114 detections, whereas the emetic gene *ces* was detected at a markedly lower frequency, since 13/114 positive samples were identified. The distribution of *B. cereus* virulence among VM product categories is shown in Figure 2A–D. The prevalence of virulence-associated genes in *B. cereus*-positive samples is shown in Table 2.

### 3.2. DNA Sequencing Confirmation of PCR Products

To further verify the identity and specificity of the PCR assays, representative amplicons obtained from the analyzed samples for all investigated target genes investigated by endpoint PCR were subjected to Sanger sequencing.

The obtained sequences were compared with reference nucleotide sequences available in the NCBI database using the BLASTn algorithm. Sequence analysis consistently confirmed the identity of the amplified PCR products. For all analyzed amplicons, the obtained sequences showed 98–100% nucleotide identity and 98–100% query coverage with *B. cereus* reference sequences (Table S1). These results provided an independent molecular confirmation of the PCR findings and supported the specificity of the amplification of the investigated *B. cereus*-associated targets.

The sequencing results were consistent across all investigated target genes and analyzed samples, providing additional evidence supporting the molecular characterization of *B. cereus* in the vending machine samples.

### 3.3. Gene Co-Occurrence and Virulence Profiles

The distribution of virulence gene combinations revealed a highly heterogeneous profile among samples. Specifically, as shown in Table 3, 27% of samples showed detection of none of the newly investigated virulence targets, whereas 14% and 16% carried one and two genes, respectively. Notably, 34% of samples showed simultaneous detection of three investigated genes, and 9% of samples exhibited simultaneous detection of all four investigated targets, representing the most extensive virulence-associated gene profile observed in the analyzed samples and indicating the presence of samples with multiple genetic markers associated with pathogenicity and persistence.

To evaluate the statistical relationship between these genes, a pairwise analysis was conducted using Fisher’s exact test, which revealed significant positive associations between specific determinants.

The most significant co-occurrence observed was between *cytK* and *hblD* (*p* < 0.001), indicating a strong positive correlation between these two enterotoxin genes. The presence of *cytK* is also statistically significantly associated with *ces* (*p* = 0.019). Remarkably, all *ces*-positive samples simultaneously showed detection of *hblD* (100%, *p* < 0.001), indicating the simultaneous detection of these two virulence-associated genetic determinants in the analyzed samples.

### 3.4. Influence of Sample Matrix on Virulence-Associated Gene Detection

A statistically significant association was observed between virulence score and sample matrix (Kruskal–Wallis test, *p* = 0.0004). Samples from milk powder showed the highest gene detection scores (mean rank = 81.95), followed by ginseng powder (71.70), whereas coffee powder exhibited the lowest values (36.33). This distribution indicates a higher frequency of simultaneous detection of the investigated genetic determinants in milk-powder samples compared with the other analyzed matrices. In particular, the proportion of samples showing simultaneous detection of ≥3 investigated genes was markedly higher in milk-derived samples compared to other matrices. The complete results of the Kruskal–Wallis test, including the number of samples and mean ranks for each sample matrix, are detailed in Table 4.

## 4. Discussion

A previous study focused on the quality and safety of VMs and documented the distribution of *B. cereus* in these environments. The present study expands this knowledge by investigating the virulence-associated gene profiles detected in the recovered samples through the characterization of key virulence-associated genes.

One of the most relevant findings was the high prevalence of the *cwpFM* gene, detected in 67% of *B. cereus*-positive samples. This gene has been associated with biofilm formation, promoting *B. cereus* adhesion and surface persistence [6,9]. These features are particularly relevant in VMs, where internal components such as narrow plastic tubing, valves, mixers, and other poorly accessible areas may provide suitable niches for biofilm establishment, especially when cleaning-in-place (CIP) procedures are insufficient [10,11]. Previous epidemiological studies have linked foodborne outbreaks caused by *B. cereus* to contamination of VM components, particularly mixing chambers and dispensing tubes [15].

The detection of enterotoxin-associated genes contributes to the evaluation of their potential pathogenicity-related and food-safety relevance. More than half of the *B. cereus*-positive samples showed detection of *cytK* (54%) and *hblD* (53%). The presence of these genes indicates genetic markers associated with enterotoxin-related functions; however, their detection does not demonstrate gene expression or toxin production. These findings are consistent with previous studies reporting the widespread distribution of enterotoxin genes within the *B. cereus sensu lato* group [4,5]. Comparable frequencies have also been described in dairy-associated isolates from Italian regions, including Apulia [16].

The *ces* gene, responsible for cereulide synthesis, was detected in 11% of *B. cereus*-positive samples, consistent with the lower prevalence of emetic strains generally reported in the literature [17]. However, detection of *ces* is relevant from a food-safety perspective because it represents a genetic marker associated with the capacity for cereulide biosynthesis, and cereulide is highly stable and resistant to heat treatment, pH variations, and proteolytic degradation [4]. Therefore, once produced in food, the toxin cannot be eliminated during beverage preparation, including hot water addition in automated dispensing systems. This aspect is particularly relevant for vulnerable populations, as highlighted by the European Food Safety Authority (EFSA) through the establishment of a highly conservative acute reference dose for infants [18]. However, the present study did not assess *ces* expression or cereulide production.

Beyond the presence of individual virulence genes, their co-occurrence was also evaluated. Approximately 60% of samples showed simultaneous detection of two or more virulence-associated genes, while 34% showed detection of three investigated genes together, generally lacking only *ces*. The most frequent combination was the simultaneous detection of *cytK* and *hblD* in 43% of samples. This association has previously been linked to increased cytotoxic activity, suggesting a possible synergistic contribution of these enterotoxins to pathogenicity [9,14]. Notably, 9% of samples showed all four investigated targets simultaneously (*cwpFM*, *cytK*, *hblD*, and *ces*), representing the most extensive virulence-associated gene profile identified in this study.

The distribution of virulence determinants also varied among the analyzed matrices, with powdered milk showing the highest prevalence of all investigated genes. This observation is consistent with the intrinsic physicochemical characteristics of this matrix, which may favor the survival and persistence of *B. cereus* [13,16] and with possible contamination during handling or storage.

A particularly relevant finding was that the *ces* gene was detected in *B. cereus*-positive DNA extracts obtained from powdered milk samples collected from 8 of the 30 (26.7%) VMs investigated, as shown in Figure 2. Although the presence of the *ces* gene does not demonstrate toxin production, this finding highlights the need for stringent microbiological monitoring, appropriate storage and handling of powdered ingredients, and effective sanitation procedures in VM systems.

In contrast, *B. cereus* was not detected in the cocoa samples analyzed in the present study. This absence may be related to matrix-specific characteristics and/or differences in handling and storage conditions. Cocoa contains polyphenolic compounds that have been reported to exhibit antimicrobial activity, which could represent a possible contributing factor, although this hypothesis cannot be established from the present data. These matrix-specific characteristics may contribute to the differences in contamination patterns observed among the analyzed vending-machine matrices [12].

The frequent simultaneous detection of multiple determinants suggests that some samples contained combinations of genes associated with environmental persistence traits and toxin-related functions.

These findings emphasize the importance of evaluating complete virulence-associated gene profiles rather than relying on single genetic markers, as the simultaneous detection of several determinants may indicate a wider pathogenicity-associated genetic profile; however, their biological activity cannot be established by PCR detection alone.

A risk-based approach is therefore essential for *B. cereus* assessment, as species-level identification alone provides limited information on the actual hazard associated with contamination. The *B. cereus* sensu lato group shows considerable variability in virulence potential, and many environmental isolates may represent background contaminants with limited clinical relevance. Molecular characterization of virulence-associated genes is consequently crucial to characterize the virulence-associated gene profiles of *B. cereus*-positive samples and to support more accurate food safety evaluations [8,13].

In the context of VMs, these findings identify several critical control points, including the microbiological quality of powdered ingredients, equipment design, and sanitation procedures. Ineffective or insufficient cleaning may facilitate biofilm persistence and reduce the effectiveness of conventional hygiene practices [11,12].

From a regulatory perspective, these findings fall within the framework of European food safety legislation, which places primary responsibility for food safety on food business operators and requires the implementation of appropriate hygiene and HACCP-based procedures [19,20]. Moreover, Regulation (EC) No 853/2004 establishes specific hygiene requirements for products of animal origin, including dairy-derived ingredients such as milk powder, identified in the present study as a relevant risk matrix [21]. Strengthened sanitation and CIP (Cleaning-In-Place) procedures, stricter monitoring of powdered ingredients, and targeted molecular surveillance alongside conventional microbiological controls are essential to reduce *B. cereus* contamination and protect consumer health.

The detection of multiple virulence-associated genes together with the biofilm-associated *cwpFM* gene may indicate the presence of *B. cereus* populations with enhanced persistence and virulence-associated genetic potential, which may persist within VMs components for extended periods and be repeatedly released into food matrices during beverage preparation.

This combination of persistence- and virulence-associated genetic markers suggests that contamination in VMs may represent a continuous rather than sporadic event.

Previous studies have also shown that, even under regular sanitation schedules, microbial contamination associated with powdered ingredients may persist and stabilize rather than being eliminated in hot beverage VMs [22]. Therefore, improving sanitation protocols and implementing strategies specifically targeting biofilm disruption should be considered key priorities for enhancing the microbiological safety of automated beverage dispensing systems.

Furthermore, when interpreting these contamination patterns, it is important to acknowledge that multiple positive samples collected from the same VM may not represent entirely independent contamination events. Instead, the detection of similar virulence-associated gene profiles across different matrices or components within the same equipment could reflect the widespread colonization and persistence of a specific *B. cereus* population throughout the machine’s internal system.

Despite providing valuable insights, this study has several limitations.

First, since multiple samples were collected from the same vending machine, the assumption of independence required for the statistical analyses may not have been fully satisfied. Therefore, the results of the statistical comparisons between matrices are exploratory and should be interpreted with caution.

Moreover, its retrospective design relying on preserved DNA extracts, without viable bacterial isolates, prevented deeper phenotypic and high-resolution genomic characterizations (e.g., whole-genome sequencing) to assess the genetic diversity and phylogeny of the *B. cereus* populations.

Although Sanger sequencing of the PCR amplicons provided independent confirmation of the identity and specificity of the molecular targets investigated, the available data do not allow strain-level characterization or phylogenetic analysis of the *B. cereus* populations present in the analyzed samples.

Furthermore, the PCR-based approach only detected the presence of selected virulence and persistence-associated genes (such as *ces*, *cytK*, and *hblD*). While this highlights a potential genetic capacity, it does not demonstrate actual gene expression or toxin production, which would be necessary for a more direct assessment of pathogenicity and health risks. In addition, the detection of virulence-associated genes in DNA extracts does not establish that the corresponding toxins were produced or present in the beverages.

Moreover, although the findings identify genetic determinants potentially relevant to food safety, the lack of quantitative data regarding bacterial and toxin concentrations, exposure levels, and consumption patterns prevented the integration of these findings into a formal quantitative food safety risk assessment model.

Finally, the study’s cross-sectional nature and geographic restriction to Campania Region in Southern Italy, limit conclusions regarding long-term bacterial persistence, contamination dynamics, and the broader generalizability of the findings. Future longitudinal studies combining viable organism isolation, quantitative toxin assays, and advanced sequencing are required to fully elucidate the persistence, diversity, and actual food safety relevance of *B. cereus* in VMs environments.

Overall, this study demonstrates that *B. cereus*-positive samples from VM environments frequently showed simultaneous detection of persistence-associated and virulence-associated genes. These findings reinforce the need to move beyond conventional species identification by incorporating molecular virulence profiling into routine surveillance programs. Such an approach would enable a more accurate distinction between environmental contaminants and strains with greater public health relevance, supporting the development of evidence-based control strategies to improve the safety of automated beverage dispensing systems.

## 5. Conclusions

The present study characterizes virulence- and persistence-associated genes in *B. cereus*-positive samples from VMs, highlighting the potential food safety implications of contamination in these environments. Notably, the detection of the *ces* gene in milk powder indicates the potential for cereulide production and further emphasizes the importance of monitoring *B. cereus* and its associated toxigenic potential in vending machine systems.

Given the limited number of studies addressing *B. cereus* in vending machine systems, further research is warranted to quantify toxin production, characterize biofilm dynamics, and assess consumer exposure and associated health risks. Such evidence will be essential to support more effective and evidence-based food safety control strategies for these increasingly widespread systems.

## Figures and Tables

**Table 1:** Primers used in the end-point PCR assay.

Gene	Primer Name	Sequences	Amplicon (bp)	Ta (°C)	References
*cwpfm*	cwpfm—fw	GGAACTGGATACGTAAGC	255	58	[14]
	cwpfm—rev	CGGCCGTTATGGTTAATTT			
*cyt-K*	cyt K—fw	TAAAGAAACGRGCGCTGTTA	81	58	
	cyt K—rev	CTGGYGCTAGTGCAACATTA			
*hblD*	hblD—fw	GCATGGTCAATTGGTGGT	163	58	
	hblD—rev	CACCAGCTGCTGTTCCTA			
*ces*	ces—fw	GGGAGCCAACAACAATGTCT	97	58	
	ces—rev	CATGCAAATGGTGTAACGATG			

**Table 2:** Prevalence of virulence genes on the total of 114 *B. cereus*-positive samples.

Gene	Positive (*n*)	Prevalence (%)
*cwpFM*	76	67
*cytK*	61	54
*hblD*	60	53
*ces*	13	11

**Table 3:** Prevalence of virulence-associated gene co-occurrence (*cwpFM*, *cytK*, *hblD*, *ces*).

Number of Genes	Prevalence (*n*/%)
0	31/27
1	16/14
2	18/16
3	39/34
4	10/9

**Table 4:** Kruskal–Wallis test results: Influence of sample matrix on virulence-associated gene detection.

Sample Matrix	*n*	Mean Rank
Milk powder	21	81.95
Ginseng	5	71.7
Coffee	12	36.33
Dispensing Nozzle (DN)	20	43.2
Water Intake Pipes (WIP)	17	58.5
Mixer bowls (MIX)	20	47.98
Cappuccino	19	64.29

## Data Availability

The original contributions presented in this study are included in the article. Further inquiries can be directed to the corresponding author. The sequences obtained from the PCR products were not deposited in GenBank, as they showed 98–100% homology with sequences already publicly available and, given the short length of the amplicons, did not provide additional information of relevance to the scientific community.

## Supplementary Materials

The following supporting information can be downloaded at: https://www.mdpi.com/article/10.3390/foods15183296/s1, Table S1: Detailed sampling scheme, PCR analysis, and virulence gene profiling of *Bacillus cereus*-positive samples.

## Abbreviations

The following abbreviations are used in this manuscript:

ARfD	Acute Reference Dose
CIP	Cleaning-in-place
DN	Dispensing Nozzle
DNA	Deoxyribonucleic acid
EFSA	European Food Safety Authority
GMP	Good Manufacturing Practices
HACCP	Hazard analysis and critical control point
MIX	Mixer bowls
PCR	Polymerase Chain Reaction
VM	Vending Machines
WIP	Water intake pipes
